# Drug Side-Effect Prediction Via Random Walk on the Signed Heterogeneous Drug Network

**DOI:** 10.3390/molecules24203668

**Published:** 2019-10-11

**Authors:** Baofang Hu, Hong Wang, Zhenmei Yu

**Affiliations:** 1 School of Data and Computer Science, Shandong Women’s University, Jinan 250014, China; 34019@sdwu.edu.cn; 2 School of Information Science and Engineering, Shandong Normal University, Jinan 250014, China; 111052@sdnu.edu.cn

**Keywords:** side-effect prediction, signed heterogeneous information network, random walk, modes of action of drugs

## Abstract

Drug side-effects have become a major public health concern as they are the underlying cause of over a million serious injuries and deaths each year. Therefore, it is of critical importance to detect side-effects as early as possible. Existing computational methods mainly utilize the drug chemical profile and the drug biological profile to predict the side-effects of a drug. In the utilized drug biological profile information, they only focus on drug–target interactions and neglect the modes of action of drugs on target proteins. In this paper, we develop a new method for predicting potential side-effects of drugs based on more comprehensive drug information in which the modes of action of drugs on target proteins are integrated. Drug information of multiple types is modeled as a signed heterogeneous information network. We propose a signed heterogeneous information network embedding framework for learning drug embeddings and predicting side-effects of drugs. We use two bias random walk procedures to obtain drug sequences and train a Skip-gram model to learn drug embeddings. We experimentally demonstrate the performance of the proposed method by comparison with state-of-the-art methods. Furthermore, the results of a case study support our hypothesis that modes of action of drugs on target proteins are meaningful in side-effect prediction.

## 1. Introduction

Drug side-effects, or adverse drug reactions (ADRs), can be regarded as undesirable effects that are caused by normal use of drugs. Most are natural pharmacological actions of the drugs and are unavoidable. Side-effects of approved drugs are harmful to patients and can even be fatal. Serious side-effects cause 100,000 deaths per year in the United States and have been one of the leading causes of death [1]. The detection of side-effects is challenging in every stage of drug development.

Traditional side-effect detection methods such as vitro safety profiling and clinical drug safety trials are time-consuming and expensive, and many potential side-effects cannot be detected because there are so many side-effect terms. Many computational methods for analyzing or predicting drug side-effects have been proposed recently [2,3,4,5]. Li et al. [6] combined multiple data sources and proposed an inductive matrix completion method for predicting unknown side-effects. Zhang et al. [7] proposed an integrative label propagation algorithm to predict potential side-effects based on high-order similarity. Zheng et al. [8] built a drug similarity integration framework to measure the similarity between drugs from various perspectives and proposed a highly reliable negative sample selection method to improve the prediction accuracy. Zhang et al. [9] adopted ensemble methods and the feature-selection-based multilabel k-nearest neighbor method (FS-MLKNN) for side-effect prediction. Liu et al. [10] built a series of binary classifiers to determine whether a drug has a specified side-effect. With the advancement of graph embedding technologies [11,12,13], many studies have focused on learning integrated drug embeddings for various prediction tasks. Ma et al. [14] proposed a drug embedding method that is based on multi-view deep autoencoders to predict drug side-effects. Hu et al. [15] proposed a heterogeneous network embedding approach by integrating PPI information into drug embeddings.

The methods that are discussed above mostly utilize chemical profiles (e.g., fingerprints) and biological profiles (e.g., target proteins, pathways, and transporters) of drugs to predict potential side-effects. It has been proved that drug–target interactions play an important role in side-effect prediction [16,17,18]. However, the drug–target interactions that are considered in these works focus only on whether a single drug acts on a target protein and neglect the modes of action of drugs on target proteins. There are various and even opposite action modes when drugs act on target proteins, such as activation, inhibition, agonist, antagonist, potentiator, blocker, inducer, suppressor, and so on. The literature [19] found that modes of action of drugs are strongly related to the therapeutic effects and side-effects of drugs through statistical analysis.

Based on the results of [19], in this paper, we propose utilizing the action mode information of drug–target interactions to learn more effective drug embeddings for drug side-effect prediction. The modes of action of drugs on target proteins can be represented as positive or negative edges in a signed graph (as shown in Table 1). When two drugs act coherently on a common target protein, the two drugs are regarded as similar and are connected with a positive edge. In contrast, when two drugs act incoherently on a common target protein, the two drugs are regarded as dissimilar and are connected with a negative edge. This produces a signed drug network. Signed networks have been frequently used in systems biology [20,21,22,23] and can reveal the deeper complex relations between individuals. The signed drug information and other traditional drug information form a signed heterogeneous information network (signed HIN). A signed HIN is a network with multiple types of nodes and links, and the links can be positive or negative [24]. Multiple types of drug information and every kind of information can be formulated as a signed subnetwork or an unsigned subnetwork. The subnetworks form a signed drug HIN. We projected every drug into a low dimensional vector space and predicted the side-effect of the drug. The low dimension vector representations of drugs synthesized different drug information. The problem of learning drug representations can be formulated as a network embedding task on the signed drug HIN. We propose utilizing random walk [25] on the signed HIN to obtain the drug sequences and utilizing the Skip-gram model [26] to learn drug embeddings. We also experimentally demonstrate that the learned drug embeddings that integrating additional signed drug information can substantially improve the side-effect prediction accuracy.

**Figure 1:**
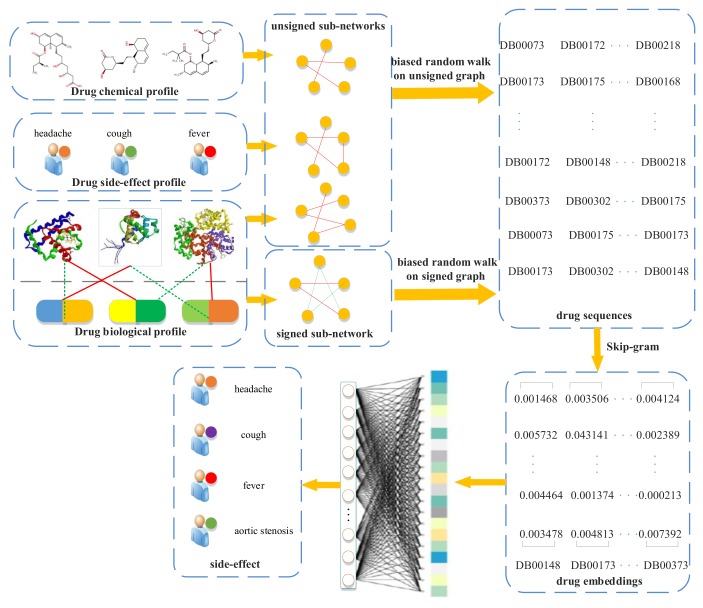
Flowchart of our side-effects prediction model (random walk on a signed heterogeneous information network (RW-SHIN)).

A flowchart of our side-effect prediction model via random walk on a signed HIN (RW-SHIN) is shown in Figure 1. First, we collected drug profiles from public databases, especially action modes of drug–target interactions, from the DrugBank database [27]. Then, we constructed a signed heterogeneous information network (signed HIN) based on these profiles. The signed HIN contained one signed drug subnetwork and three unsigned drug subnetworks. RW-SHIN performed different random walk procedures on signed subnetworks versus unsigned subnetworks. Here, two biased random walk procedures [28,29], which can effectively incorporate positive and negative relations into a graph, were used to obtain nodes sequences. Then, we utilized the Skip-gram model to learn drug embeddings based on the node sequences. After that, the drug embeddings were input into a fully connected neural network to predict a potential side-effect.

## 2. Materials

### 2.1. Drug Chemical Profile

We collected the fingerprints of drugs from the PubChem Compound database [30]. Each drug was encoded into an 881-dimensional feature vector, with entries of 0 or 1 representing the presence or absence, respectively, of a chemical substructure term.

### 2.2. Drug Side-Effect Profile

**Figure 2:**
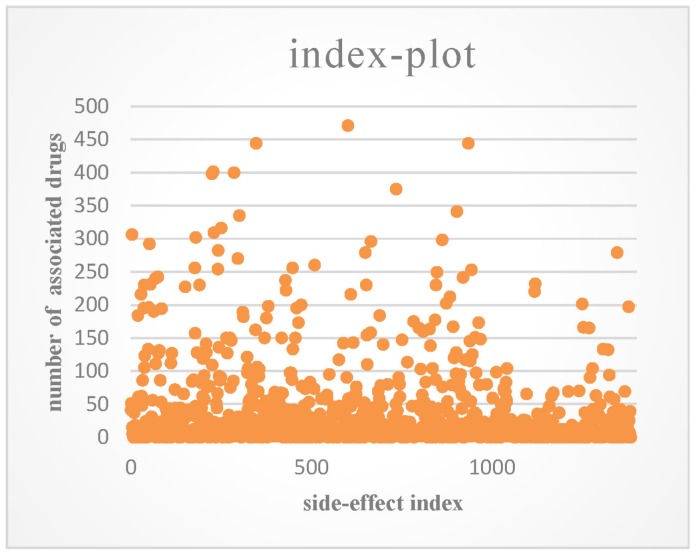
Index plot of the number of associated drugs for each side-effect.

SIDER [31] is a database that contains the side-effects of drugs on the market that are listed in their package inserts. We downloaded the entire database from http://sideeffects.embl.de/. The information in SIDER is limited because many side-effects may not be detected in clinical trials. OFFSIDES [32] is another side-effect database, which was built by mining the FDA Adverse Event Reporting System (FAERS, http://www.fda.gov/cder/aers/default.htm). We extracted the associations between drugs and side-effects from the two databases. This produced a dataset that consisted of 548 drugs, 1385 side-effect terms, and 41,008 associations between drugs and side-effects, and each drug had 74.8 side-effects on average. An index plot of the number of associated drugs for each side-effect is shown in Figure 2.

Each drug was represented by a 1385-dimensional feature vector, where each element is 1 or 0, which represents the presence or absence, respectively, of a side-effect term for this drug.

### 2.3. Drug Biological Profile

We crawled the biological profiles of drugs in the DrugBank database [27], which is a widely used public drug information database. We extracted the target proteins of the 548 drugs without considering the action modes of the drugs on the target proteins. Each drug is encoded into a 780-dimensional feature vector, with 0 or 1 representing the presence or absence, respectively, of a target protein for this drug.

Meanwhile, we crawled the action modes of 548 drugs on 780 target proteins and associated every mode with a sign according the procedure that is described in [15] (as presented in Table 1). This produced a signed drug–target association matrix $A\in {\left\{-1,0,1\right\}}^{m*n}$, where *m* is the number of drugs and *n* is the number of targets.

### 2.4. Construction of the Signed Drug Heterogeneous Information Network

We use the above three profiles to construct a drug heterogeneous information network (HIN) and learn the drug embeddings based on the constructed drug HIN. A heterogeneous information network is a special type of information network that contains several types of nodes or links. In this work, the drug HIN contains three unsigned subnetworks and one signed drug subnetwork.

#### 2.4.1. Unsigned Drug Subnetworks

We constructed three unsigned drug subnetworks based on drug chemical profiles, drug side-effect profiles and unsigned drug–target associations in the drug biological profiles. The nodes in the three subnetworks correspond to all 548 drugs. The edges are defined by the Jaccard similarities of the feature vectors in every type of profile. The edge weight $w_{ij}^{u}$ between drug pair $\left(d_{i},d_{j}\right)$ in an unsigned subnetwork is defined by Equation (1).

(1) $$w_{ij}^{u}=\frac{\left|Pr_{i}\cap Pr_{j}\right|}{\left|Pr_{i}\cup Pr_{j}\right|}$$

where $Pr_{i}$ is the feature vector of drug *i* in a type of drug profile.

#### 2.4.2. Signed Drug Subnetwork

**Figure 3:**
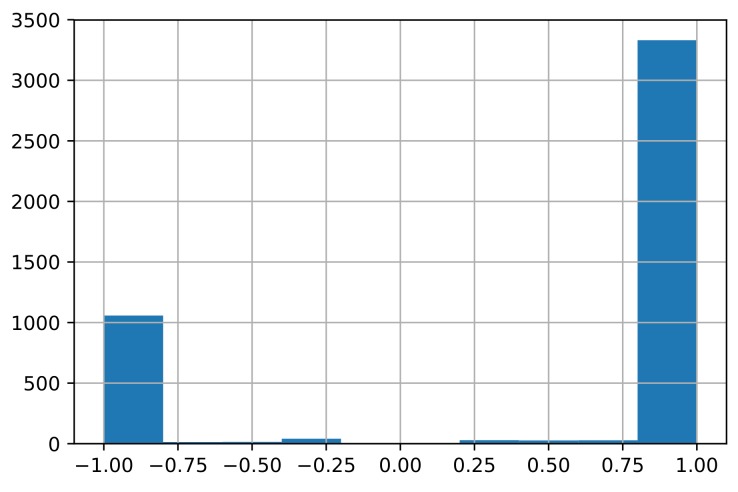
Distribution of the edge weights $w_{ij}^{s}$. (The number of edges is 4544. The number of negative edges with weights $w_{ij}^{s}<-0.5$ is 1074, while the number of positive edges with weights $w_{ij}^{s}>0.5$ is 3369.)

We constructed a signed drug subnetwork $G^{s}$ using the signed drug–target association matrix $A\in {\left\{-1,0,1\right\}}^{m*n}$. The node set in $G^{s}$ is the drug set and the edge weight $w_{ij}^{s}$ between drug pair $\left(d_{i},d_{j}\right)$ is defined by Equation (2).

(2) $$w_{ij}^{s}=\frac{\sum_{k=1}^{n}a_{ik}{\cdot a}_{jk}}{\sum_{k=1}^{n}\left|a_{ik}{\cdot a}_{jk}\right|}$$

where $a_{ik}$ is the element of the signed drug–target association matrix *A*. $w_{ij}^{s}$ ranges from −1 to 1, where $w_{ij}^{s}=-1$ if drug pair $\left(d_{i},d_{j}\right)$ share several targets but have opposite action modes on the shared targets, while $w_{ij}^{s}=1$ if drug pair $\left(d_{i},d_{j}\right)$ share several targets and have the same action mode on the shared targets and $w_{ij}^{s}=0$ if drug pair $\left(d_{i},d_{j}\right)$ have no shared targets. The distribution of the edge weights $w_{ij}^{s}$ is plotted in Figure 3.

**Figure 4:**
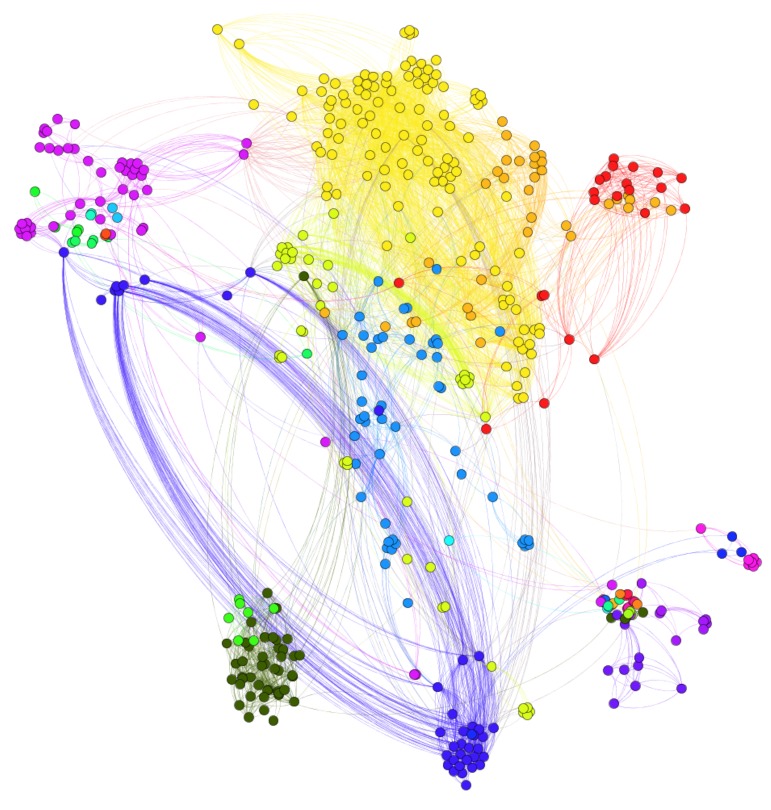
Signed drug sub-network. (Nodes are drugs and the number of edges is 4544. There are 3416 positive edges and 1128 negative edges. Lines in red color represent positive links while lines in green color represent negative links.)

According to Figure 3, most drug pairs have strong connections for both positive and negative edges. Hence, the drug pairs have only several targets, but the actions on the shared targets are extremely similar or different. The side-effects may differ if the actions on the shared targets differ. The signed drug subnetwork is illustrated in Figure 4.

## 3. Methods

In our model, side-effects were predicted using the low dimensional vector representations of drugs. The drug representations are learned from the above-mentioned drug profiles. Learning comprehensive drug representations can be formulated as the task of learning node embeddings of the constructed drug HIN. Therefore, we first performed a random walk procedure on every subnetwork in the above-constructed drug HIN to obtain nodes sequences, and then utilized the Skip-gram model to learn drug embeddings based on the obtained node sequences. After that, potential side-effects were predicted using the learned drug embeddings.

### 3.1. Random Walk on the Signed HIN

The drug HIN that is presented above contains both unsigned subnetworks and signed subnetworks. In the signed subnetworks, the learned node representations must integrate the positive and negative relations. An effective approach is to conduct random walks on the graph to integrate the positive and negative relations into node sequences. In this paper, we use two types of biased random walk procedures [28,29] to obtain many node sequences in each subnetwork. The two biased random walk procedures are conducted on signed graphs versus unsigned graphs.

#### 3.1.1. Biased Random Walk on a Signed Graph

**Figure 5:**
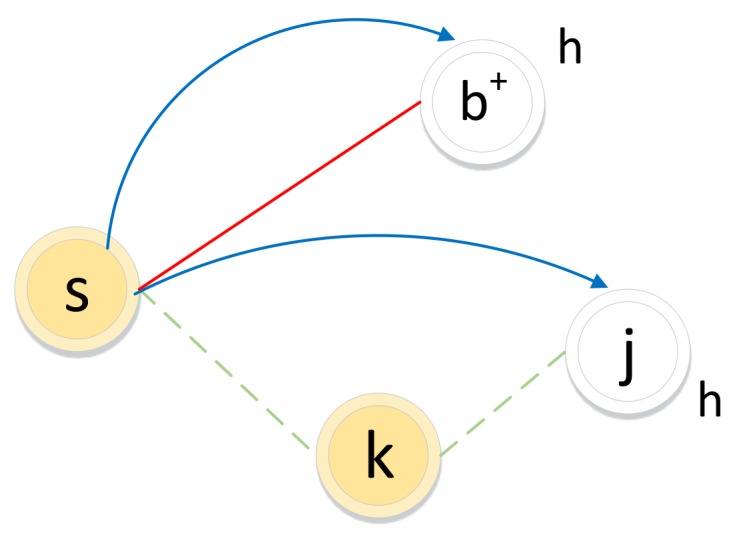
Illustration of the biased random walk procedure in a signed graph. (The walker is now resting on node *s* and evaluating its next node *h*. Green dotted lines and red solid lines represent negative edges and positive edges, respectively. Blue lines with arrows indicate the next-hop node *h*.)

The biased random walk procedure on a signed graph utilizes the direct neighbor relations and common neighbor relations to acquire a node sequence. As illustrated in Figure 5, for a random walker, let $t_{i-1}=s$ denote the i−1th node in the path and let node $t_{i}=h$ be the next-hop node.

If the edge between node *s* and node *h* is positive, the walker chooses *h* as the next hop according to the direct neighbor relation ($s\to b^{+}$) with transition probability $\frac{w_{sh}^{s}}{\sum_{i\in nbs(s)}\left|w_{si}^{s}\right|}$.

**Figure 6:**
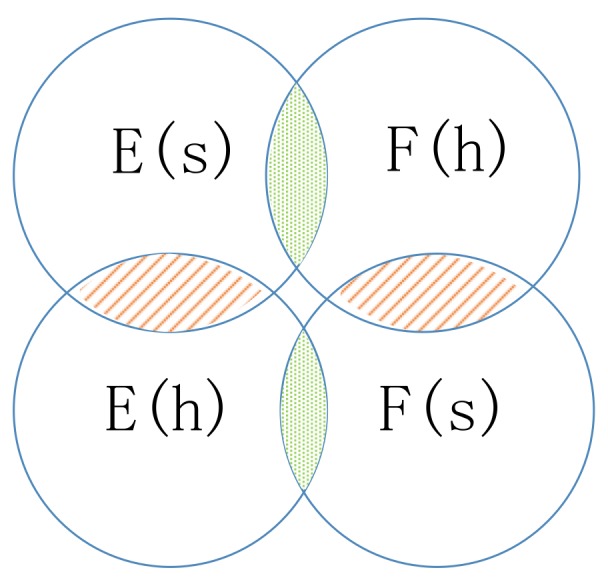
Illustration of the enemy sets and friend sets. (The overlap area is the intersection of sets.)

If the edge between node *s* and node *k* is negative, the walker will not choose *k* as the next hop but may choose $k^{\prime }s$ enemy node *j* as the next hop, which is guided by the common neighbor relation. The common neighbor relation involves two types of sets: friend sets F(w) and enemy sets E(w) (as illustrated in Figure 6). If two nodes’ friend sets or enemy sets have a large overlap area, the two nodes share common friends or enemies, respectively, and the relation between them tends to be positive. In contrast, if the enemy set of a node covers a large overlap area of the friend set of another node, the two nodes have very little in common and the relation between them will be weak or even negative.

Here, we define a common neighbor relation similarity ls(s,h), which is used to decide whether the walker jumps to an enemy’s enemy node.

(3) $$ls\left(s,h\right)=\sum_{r\in F(s)\cap F(h)}w_{sr}^{s}+\sum_{n\in E(s)\cap F(h)}w_{sn}^{s}-\sum_{m\in E(s)\cap F(h)}w_{sm}^{s}-\sum_{p\in F(s)\cap E(h)}w_{sp}^{s}$$

If ls(s,h)>0, *h* is the enemy’s enemy of node *s*. The walker will jump to *h* from *s* according to the transition probability $\frac{\left|w_{sk}^{s}\right|\cdot \left|w_{kh}^{s}\right|}{\sum_{i\in nbs(s)}\left|w_{si}^{s}\right|\cdot \sum_{j\in negativenbs(k)}\left|w_{kj}^{s}\right|}$.

The transition probability in the biased random walk procedure is defined in Equation (4).

(4) $$p_{sh}=\left\{\begin{array}{cc} p\cdot \frac{w_{sh}^{s}}{\sum_{i\in nbs(s)}\left|w_{si}^{s}\right|} & w_{sh}^{s}>0\\ q\cdot \frac{\left|w_{sk}^{s}\right|\cdot \left|w_{kh}^{s}\right|}{\sum_{i\in nbs(s)}\left|w_{si}^{s}\right|\cdot \sum_{j\in negativenbs(k)}\left|w_{kj}^{s}\right|} & w_{sk}^{s}<0_{}\;and\;w_{kh}^{s}<0_{}\;and\;w_{sh}^{s}=0_{}\;and\;ls\left(s,h\right)>0\\ 0 & otherwise \end{array}\right.$$

where *p* and *q* are adjustable parameters that guide the walker by selecting one of its friends or an enemy of its enemy as the next hop.

The biased random walk procedure assumes that an enemy’s enemy may be a friend. We investigated the triangle relationships in the signed drug subnetwork to determine whether this assumption holds in our datasets. The results are presented in Table 2.

In the above investigation, we find that an enemy’s enemy is more likely to be a friend in a signed drug subnetwork. Therefore, we can use the biased random walk procedure to combine the positive and negative relations and to obtain node sequences.

#### 3.1.2. Biased Random Walk Procedure on an Unsigned Graph

**Figure 7:**
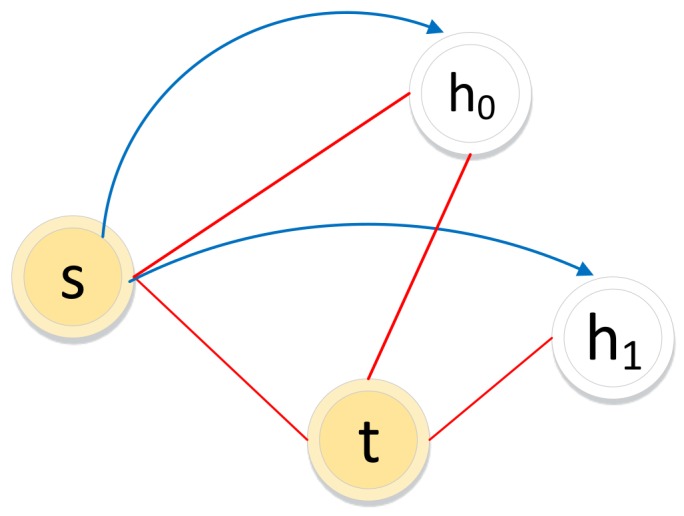
Illustration of the biased random walk procedure in an unsigned graph. (The walker is now resting on node *s* and evaluating its next node *h*. *t* is the last step in the path. Red solid lines represent edges in the graph. Blue lines with arrows represent the next possible steps.)

The biased random walk procedure on an unsigned graph combines two sampling strategies: breadth-first sampling (BFS) and depth-first sampling (DFS). For a random walker, let $t_{i-1}=s$ denote the (i−1)th node and let node $t_{i-2}=t$ denote the (i−2)th node in the path. Node $t_{i}=h$ will be the next-hop node. As illustrated in Figure 7, h0 or h1 may be the next-hop node according to BFS and DFS, respectively.

The transition probability in the biased random walk procedure is defined in Equation (5).

(5) $$p_{sh}=\left\{\begin{array}{cc} \alpha \cdot \frac{w_{sh}^{u}}{\sum_{i\in nbs(s)}w_{si}^{u}} & w_{th}^{u}\neq 0\\ \beta \cdot \frac{w_{sh}^{u}}{\sum_{i\in nbs(s)}w_{si}^{u}} & w_{th}^{u}=0 \end{array}\right.$$

where α and β are adjustable parameters that guide the walker selecting the next node following BFS or DFS. *t* is the last node in the path.

### 3.2. Learning Drug Embeddings

**Figure 8:**
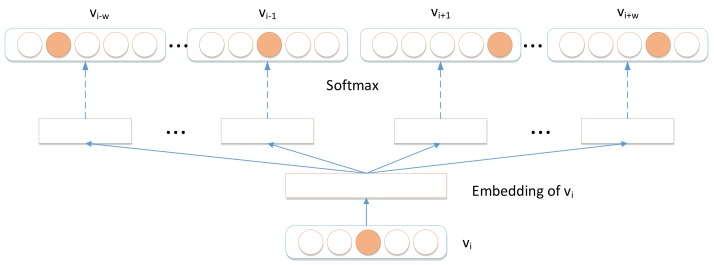
Skip-gram model.

After obtaining node sequences in the signed HIN, we utilized Skip-gram model [26] to learn node representations. The model of Skip-gram is illustrated in Figure 8 and the optimizer objective function of Skip-gram is presented as Equation (6).

(6) $$\Gamma =\min_{f}-\log\mathrm{Pr}\left(\left\{v_{i-w},\cdots ,v_{i-1},v_{i+1},\cdots ,v_{i+w}\right\}\left|f(v_{i})\right.\right)$$

where *f* is the node mapping function $f:v\in V\to R^{\left|V\right|\times d}$, in which *d* is the dimension of the embeddings; $v_{i-w}$ is the w−th neighbor node of node $v_{i}$ in every node sequence; and *w* is the window size.

### 3.3. Prediction Formulation

Once we have obtained the final drug embeddings, we use a fully connected neural network to predict each side-effect, as illustrated the last step in Figure 1. The predictive problem was modeled as a binary classification of each side-effect. For each side-effect, the drugs that are known to cause the side-effect were labeled as positive samples and the remaining drugs were labeled as negative samples. The predictive model utilized the L2-norm-regularized logistic regression method [33] to predict each side-effect. The input features of the model were drug embeddings. The loss function is presented as Equation (7).

(7) $$L\left(Z,y,w\right)=\sum_{i}Cost\left(y_{i},f\left(z_{i}*w+b\right)\right)+\lambda {∥w∥}_{2}^{2}$$

where *Z* is the feature matrix of the drugs and the row vector $z_{i}$ of *Z* is the embedding of drug *i*; *f* is a nonlinear logistic function with L2 loss; *w* and *b* are the weight vector and the bias vector, respectively; and λ is a hyperparameter, which must be learnt from the data.

## 4. Results and Discussions

We experimentally compared the performance of the proposed method with several state-of-the-art methods and utilized a case study to support our hypothesis that modes of action of drugs on target proteins are meaningful in side-effect prediction. We also discussed the impact of embedding dimension on the prediction performance to find the best representations of drugs.

### 4.1. Performance Evaluation Metrics

We used the receiver operating characteristic curve (ROC curve) to evaluate the performance of each method. The ROC curve is the true-positive rate (TPR) as a function of the false-positive rate (FPR), which is based on various thresholds, where TPR and FPR are defined in Equations (8) and (9).

(8) $$TPR=\frac{TP}{TP+FN}$$

(9) $$FPR=\frac{FP}{FP+TN}$$

where TP, FP, TN, and FN are the numbers of true positives, false positives, true negatives, and false negatives, respectively.

We calculated the area under the ROC curve (AUROC) to evaluate the performance of each method in predicting all side-effects.

### 4.2. Baselines

We compared our method (RW-SHIN) with several state-of-the-art network embedding algorithms for side-effect prediction. We implemented the following four baselines for comparison:

- Laplacian eigenmaps [34]: Laplacian eigenmaps is a typical matrix factorization method that has been widely adopted for data analysis of biomedical networks. It aims at factorizing a data matrix of a graph into lower dimensional matrices while preserving the topological properties of the original graph. For the drug HIN, we concatenated the Laplacian eigenmaps of each unsigned subnetwork to construct feature vectors of drugs for side-effect prediction.
- GCN [35]: GCN is a recently proposed network embedding method that is based on the spectral convolutional operation and realizes state-of-the-art performance on important prediction problems in recommender systems. Here, we linearly integrated the similarity matrices of the unsigned subnetworks in the drug HIN and learned the drug embeddings using GCN.
- AttSemiGAE [14]: AttSemiGAE utilizes multiview graph autoencoders (GAEs) and adds an attentive mechanism for determining the weights for each view with respect to the corresponding prediction tasks. Here, each unsigned subnetwork in the drug HIN is regarded as a single-view graph in the AttSemiGAE algorithm and the supervised information is the known side-effects of the drugs.
- RW-HIN: For further validation of the impacts of action modes on the quality of side-effect predictions, we designed a network embedding algorithm that ignores the signed drug information, namely, RW-HIN. The algorithm is based on random walk on an unsigned graph.

These algorithms cannot learn node embeddings on signed graphs and only utilize the following data sources to learn drug embeddings: drug chemical profiles, drug side-effect profiles and unsigned drug biological profiles. We performed the following 5-fold cross-validation procedure for each algorithm: The known drug–side-effect associations are used as a gold-standard set. The drugs in the gold-standard set are split into five equally sized subsets, and each subset is used in turn as the test set, while the remaining four subsets are used as the training set. The final performance is evaluated according to the results on all 5 folds. For accurate comparison, we use the same experimental conditions, namely, the same training drugs and test drugs are used across all methods in each cross-validation fold. The embedding dimension of each algorithm is 32. All parameters in each method (e.g., the regularization parameters, the adjustable parameters, and the embedding dimension) were optimized using grid search with the AUROC score as an objective function.

### 4.3. Result of Comparison

**Figure 9:**
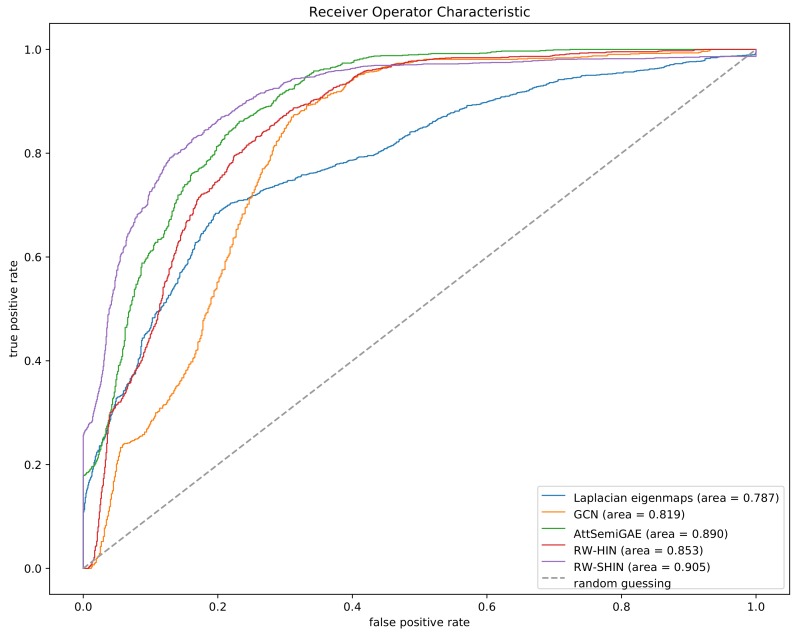
Receiver operator characteristic (ROC) curves based on the 5-fold cross-validation experiment.

Figure 9 plots the ROC curves for the five approaches based on the cross-validation experiment. The ROC curve of each method is based on the merged prediction scores of all side-effects. Our method outperforms the other methods, which suggests that the proposed method is more effective than the previous methods. Although RW-SHIN and RW-HIN are based on the same training model, RW-SHIN, which considers the modes of action of drugs on target proteins, outperforms RW-HIN. This suggests that the modes of action of drugs on target proteins are meaningful. In addition, the method with an attentive mechanism outperforms the methods that do not use attentive mechanisms on the same unsigned datasets.

**Figure 10:**
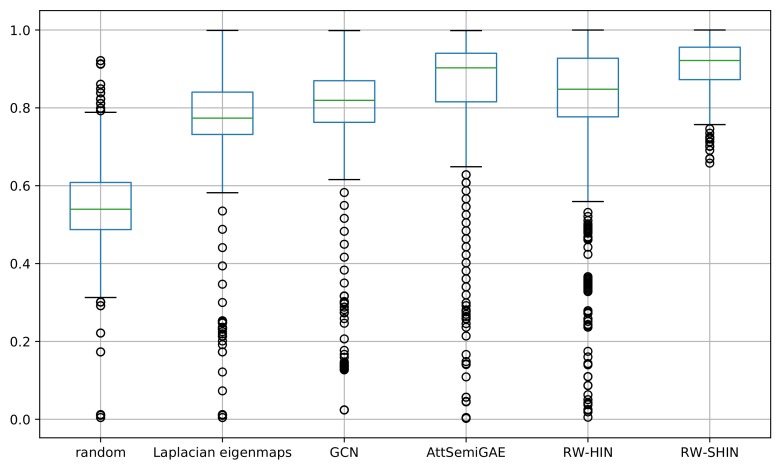
Boxplots of the area under the ROC curve (AUROC) scores for every side-effect.

We examined the prediction accuracies for individual side-effects. We calculated the AUROC scores for each side-effect. Figure 10 presents a boxplot that represents the distribution of the resulting AUROC scores for 1385 side-effects for each method. Our signed drug–target information-based method produced the best results. Most high-frequency side-effects (e.g., pseudomembranous colitis, gynecomastia and interstitial nephritis) were predicted with higher accuracy, while most low-frequency side-effects (e.g., diabetic neuropathy, nephrogenic diabetes insipidus and narcolepsy) were predicted with lower accuracy. The ROC-AUC scores are positively correlated with the side-effect frequencies. However, this is not absolute: some high-frequency side-effects (e.g., abdominal pain) were predicted with lower accuracy, while some low-frequency side-effects (e.g., chronic active hepatitis) were predicted with higher accuracy.

We also investigated the side-effect prediction accuracy for every drug by evaluating the Top−K prediction accuracy of each drug. We counted the number of drugs whose Top−1, Top−3, and Top−5 prediction results contain at least one known side-effect. If at least one known side-effect is in the Top−K prediction results of a drug, the drug will be counted. The results are presented in Table 3. The proposed signed drug–target association-based method outperforms other methods. The number of drugs whose Top−1 prediction side-effects are known is 276 (50.3%). The numbers of drugs whose Top−3 and Top−5 prediction results contain at least one known side-effect are 354 (64.6%) and 465 (84.9%), respectively.

### 4.4. Case Studies

We investigated the side-effect prediction results of drugs that are similar in terms of chemical profiles and target proteins but opposite in terms of modes of action on target proteins. We consider Betaxolol (DB00195) and Dobutamine (DB00841) as examples. Betaxolol is a selective beta-1 adrenergic receptor blocker that is used in the treatment of hypertension and glaucoma. Dobutamine is a sympathomimetic drug that is used in the treatment of heart failure and cardiogenic shock. Its primary mechanism is direct stimulation of beta-1 receptors of the sympathetic nervous system.

The chemical similarity of drugs can be calculated using Jaccard similarity, which is shown in Equation (1). The chemical similarity of the two drugs is 0.82. The two drugs have two common target proteins: Beta-1 adrenergic receptor (P08588) and Beta-2 adrenergic receptor (P07550). The unsigned drug biological similarity of the two drugs is 0.67. However, the action modes of the two drugs are opposite. Dobutamine acts as an agonist on the two target proteins, while Betaxolol acts as an antagonist. The top-10 prediction results of the two drugs are presented in Table 4.

The prediction results of the two drugs differed substantially and there was only one common side-effect: nausea. Nausea is a very common side-effect and may be caused by most drugs. This difference is consistent with the known side-effect similarity of the two drugs, which is 0.083. Focusing on the false prediction results of Dobutamine, we found that insomnia is a known side-effect of Pindolol (DB00960), which is an oral beta blocker that is used to treat hypertension. Dobutamine and Pindolol have the same action modes on the common target proteins: the Beta-1 and Beta-2 adrenergic receptors. The chemical similarity of Dobutamine and Pindolol is 0.873. Dobutamine [36] belongs to a class of beta agonists that typically have mild to moderate adverse effects, which include increased heart rate and insomnia.

### 4.5. Performance Comparison among Embedding Dimensions

**Figure 11:**
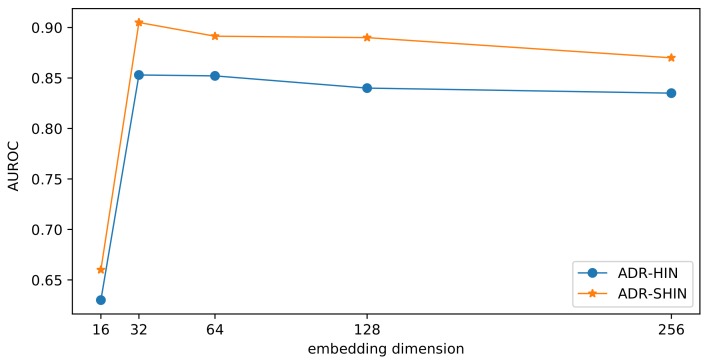
Impact of the embedding dimension on the prediction performance.

To examine the impact of the embedding size on the prediction performance, we compared our two models with various dimensions of drug embeddings in terms of AUROC. The results are presented in Figure 11. With the increase of the embedding dimension, the side-effect prediction performance initially increased and subsequently decreased. If the embedding dimension is small, the embeddings may lose too much information. If the embedding dimension is very large, the embeddings may incorporate redundant information and tend to overfit. The two methods performed optimally when the embedding dimension was 32. RW-SHIN outperformed RW-HIN at the same embedding dimensions. This supports our assumption that the modes of action of drugs on target proteins can provide useful information for drug embeddings.

## 5. Conclusions and Future Work

In this work, we proposed an improved drug side-effect prediction method by integrating modes of action of drugs on target proteins into drug information. We proposed a side-effect prediction method that learns more comprehensive drug embeddings based on drug chemical profiles, unsigned drug–target protein associations, signed drug–target protein associations, and drug side-effect profiles. To the best of our knowledge, no previous work considers modes of action of drugs on target proteins in the context of drug side-effect prediction. To formulate this feature of drug information, identical or opposite action modes were defined as positive or negative edges in a signed graph and formed a signed heterogeneous information network with other drug information. We used two bias random walk procedures to obtain drug sequences in the signed HIN and utilized the Skip-gram framework to learn drug embeddings based on the obtained drug sequences. In the cross-validation experiments, all results demonstrate that the prediction performance improved substantially using our method. According to the case study on the drugs Betaxolol and Dobutamine, our method can predict both existing and novel drug–side-effect associations. Moreover, the results of the case study support our hypothesis that action modes are meaningful in side-effect prediction.

In the model, chemical structures, target proteins, action modes, and known side-effects were integrated into a unified framework for learning drug embeddings for side-effect prediction. However, sometimes the modes of action of drugs on target proteins are not always available and complete for all drugs. It has been proven that side-effects of drugs are related to the action modes. Consequently, in our future work, we will utilize known side-effects to predict modes of action of drugs and to further enrich the action mode information. We will also extend the datasets of drugs by mining from electronic medical records [37].

## Figures and Tables

**Table 1:** Modes of action of drugs and the corresponding edge signs [15].

Edge Sign	Action Modes in DrugBank
Positive (+)	agonist; partial agonist; activator; stimulator; inducer; positive allosteric modulator; potentiator; positive modulator
Negative (−)	Inhibitor; inhibitory allosteric modulator; inhibitor competitive; antagonist; partial antagonist; negative modulator; inverse agonist; blocker; suppressor; desensitize the target; neutralizer; reducer
Not classifiable (0)	antibody; cofactor; modulator; binder; chaperone; cleavage; metabolizer; ligand; product of; component of; chelator; cross-linking/alkylation; intercalation; adduct; acetylation; allosteric modulator

**Table 2:** Number of triads in the signed drug subnetwork.

Number of Triads	Number of T1 (+ + +)	Number of T2 (− − +)	Number of T3 (+ + −)	Number of T4 (− − −)
33,470	19,700 (58.86%)	12,266 (36.65%)	1216 (3.63%)	288 (0.86%)

**Table 3:** Numbers of drugs whose top−K prediction results contain one known side-effect. (Bold values are the best prediction results.)

Method	Top−1	Top−3	Top−5
random	20 ± 2.48	52 ± 3.46	97 ± 3.44
Laplacian eigenmaps	228 ± 8.08	316 ± 7.23	429 ± 6.45
GCN	249 ± 7.28	343 ±6.39	441 ± 5.27
AttSemiGAE	264 ± 4.79	**365 ± 5.81**	460± 6.42
RW-HIN	258 ±8.12	328 ± 7.47	454 ± 7.18
RW-SHIN	**276 ±5.48**	354 ± 6.32	**465 ± 8.93**

**Table 4:** Prediction of the op−10 side-effects of Betaxolol and Dobutamine based on RW-SHIN.

Top−K	Betaxolol	Confirmed	Dobutamine	Confirmed
K=1	dysphasia	yes	hypokalemia	yes
K=2	perforated gastric ulcer	no	sneezing	no
K=3	nausea	yes	pruritus	yes
K=4	headache	yes	nausea	yes
K=5	mood disorders	yes	insomnia	no
K=6	shoulder pain	no	streptococcal pharyngitis	no
K=7	hypercholesterolemia	yes	heartburn	yes
K=8	otitis externa	yes	skin necrosis	yes
K=9	tumor	no	adrenal disease	no
K=10	ear pain	yes	dyskinesia	no

## References

[1] Giacomini K.M., Krauss R.M., Dan M.R., Eichelbaum M., Hayden M.R., Nakamura Y. (2007). When good drugs go bad. Nature.

[2] Yamanishi Y., Pauwels E., Kotera M. (2012). Drug side-effect prediction based on the integration of chemical and biological spaces. J. Chem. Inf. Model..

[3] Li J., Zheng S., Chen B., Butte A.J., Swamidass S.J., Lu Z. (2015). A survey of current trends in computational drug repositioning. Brief. Bioinform..

[4] Xu B., Shi X., Zhao Z., Zheng W. (2018). Leveraging biomedical resources in bi-lstm for drug–drug interaction extraction. IEEE Access.

[5] Vilar S., Tatonetti N.P., Hripcsak G. (2015). 3D pharmacophoric similarity improves multi adverse drug event identification in pharmacovigilance. Sci. Rep..

[6] Rong L., Dong Y., Kuang Q., Wu Y., Li Y., Min Z., Li M. (2015). Inductive matrix completion for predicting adverse drug reactions (adrs) integrating drug–target interactions. Chemom. Intell. Lab. Syst..

[7] Zhang P., Wang F., Hu J., Sorrentino R. (2015). Label propagation prediction of drug–drug interactions based on clinical side effects. Sci. Rep..

[8] Zheng Y., Peng H., Ghosh S., Lan C., Li J. (2019). Inverse similarity and reliable negative samples for drug side-effect prediction. BMC Bioinform..

[9] Zhang W., Liu F., Luo L., Zhang J. (2015). Predicting drug side effects by multi-label learning and ensemble learning. BMC Bioinform..

[10] Liu M., Wu Y., Chen Y., Sun J., Zhao Z., Chen X.W., Matheny M.E., Xu H. (2012). Large-scale prediction of adverse drug reactions using chemical, biological, and phenotypic properties of drugs. J. Am. Med. Inform. Assoc..

[11] Yan S., Xu D., Zhang B., Zhang H.J., Yang Q., Lin S. (2006). Graph embedding and extensions: A general framework for dimensionality reduction. IEEE Trans. Pattern Anal. Mach. Intell..

[12] Cao S. Deep Neural Network foR Learning Graph Representations. Proceedings of the Thirtieth Aaai Conference on Artificial Intelligence.

[13] Huang Z., Mamoulis N. (2017). Heterogeneous information network embedding for meta path based proximity. arXiv.

[14] Ma T., Xiao C., Zhou J., Wang F. (2018). Drug similarity integration through attentive multi-view graph auto-encoders. arXiv.

[15] Hu B., Wang H., Wang L., Yuan W. (2018). Adverse Drug Reaction Predictions Using Stacking Deep Heterogeneous Information Network Embedding Approach. Molecules.

[16] Campillos M., Kuhn M., Gavin A.C., Jensen L.J., Bork P. (2008). Drug target identification using side-effect similarity. Science.

[17] Mizutani S., Pauwels E., Stoven V., Goto S., Yamanishi Y. (2012). Relating drug–protein interaction network with drug side effects. Bioinformatics.

[18] Yamanishi Y., Kotera M., Moriya Y., Sawada R., Goto S. (2014). Dinies: Drug–target interaction network inference engine based on supervised analysis. Nucleic Acids Res..

[19] Torres N.B., Altafini C. (2015). Drug combinatorics and side effect estimation on the signed human drug–target network. BMC Syst. Biol..

[20] Iacono G., Altafini C. (2010). Monotonicity, frustration, and ordered response: An analysis of the energy landscape of perturbed large-scale biological networks. BMC Syst. Biol..

[21] Iacono G., Ramezani F., Soranzo N., Altafini C. (2010). Determining the distance to monotonicity of a biological network: A graph-theoretical approach. IET Syst. Biol..

[22] Milo R., Shen-Orr S., Itzkovitz S., Kashtan N., Chklovskii D., Alon U. (2002). Network motifs: Simple building blocks of complex networks. Science.

[23] Sontag E.D. (2007). Monotone and near-monotone biochemical networks. Syst. Synth. Biol..

[24] Wang H., Zhang F., Min H., Xing X., Guo M., Qi L. (2017). Shine: Signed heterogeneous information network embedding for sentiment link prediction. arXiv.

[25] Lovász L. (1993). Random walks on graphs: A survey. Combinatorics, Paul Erdos Is Eighty.

[26] Mikolov T., Sutskever I., Chen K., Corrado G., Dean J. (2013). Distributed representations of words and phrases and their compositionality. Adv. Neural Inf. Process. Syst..

[27] Knox C., Law V., Jewison T., Liu P., Wishart D.S. (2010). Drugbank 3.0: A comprehensive resource for ‘omics’ research on drugs. Nucleic Acids Res..

[28] Hu B., Wang H., Yu X., Yuan W., He T. (2019). Sparse network embedding for community detection and sign prediction in signed social networks. J. Ambient Intell. Hum. Comput..

[29] Grover A., Leskovec J. (2016). Node2vec: Scalable feature learning for networks. arXiv.

[30] Wang Y., Xiao J., Suzek T.O., Zhang J., Wang J., Bryant S.H. (2009). Pubchem: A public information system for analyzing bioactivities of small molecules. Nucleic Acids Res..

[31] Kuhn M., Campillos M., Letunic I., Jensen L.J., Bork P. (2010). A side effect resource to capture phenotypic effects of drugs. Mol. Syst. Biol..

[32] Tatonetti N.P., Ye P.P., Daneshjou R., Altman R.B. (2012). Data-driven prediction of drug effects and interactions. Sci. Transl. Med..

[33] Hastie T., Tibshirani R., Friedman J., Franklin J. (2005). The elements of statistical learning: Data mining, inference and prediction. Math. Intell..

[34] Belkin M. (2002). Laplacian eigenmaps and spactral techniques for embedding and clustering. Adv. Neural Inf. Process. Syst..

[35] Kipf T.N., Welling M. (2016). Semi-supervised classification with graph convolutional networks. arXiv.

[36] Ishide T. (2002). Denopamine, a selective beta1-receptor agonist and a new coronary vasodilator. Curr. Med. Res. Opin..

[37] Wang L., Wang H., Song Y., Wang Q. (2019). MCPL-Based FT-LSTM: Medical Representation Learning-Based Clinical Prediction Model for Time Series Events. IEEE Accesss.

